# Case report: Disseminated histoplasmosis in a renal transplant recipient from a non-endemic region

**DOI:** 10.3389/fped.2022.985475

**Published:** 2022-11-14

**Authors:** Brian Chang, Tawny Saleh, Cameron Wales, Lawrence Kuklinski, Prerana Malla, Shangxin Yang, David Fuller, Karin Nielsen-Saines

**Affiliations:** ^1^ Department of Pediatrics, UCLA Mattel Children's Hospital, Los Angeles, CA, United States; ^2^ Department of Pediatrics, Pediatric Infectious Disease Division, UCLA Mattel Children's Hospital, Los Angeles, CA, United States; ^3^ UCLA Pathology and Laboratory Medicine, Ronald Reagan UCLA Medical Center, Los Angeles, CA, United States; ^4^ Department of Medicine, Infectious Diseases Division, Ronald Reagan UCLA Medical Center, Los Angeles, CA, United States

**Keywords:** disseminated histoplasmosis, pediatric, renal transplant, immunosuppression, pathology

## Abstract

Histoplasmosis is the most common endemic fungal infection in the USA. The majority of cases are asymptomatic and have clear exposure to endemic regions. In contrast, we present an adolescent immunocompromised patient with systemic and relatively non-specific symptoms including abdominal pain, weight loss, lower extremity edema, and scabbing skin lesions, without known exposure to endemic areas for histoplasmosis. Histologic analysis of gastrointestinal and skin biopsies eventually revealed a diagnosis of disseminated histoplasmosis; the patient was successfully treated with amphotericin B followed by itraconazole maintenance therapy. Ultimately, a high bar of suspicion for fungal disease must be maintained in immunosuppressed individuals even without apparent exposure history to endemic areas. This case report serves as a valuable reference for practitioners evaluating differential diagnosis of infections in immunocompromised patients.

## Introduction

Histoplasmosis is a relatively widespread fungal disease and is often asymptomatic in immunocompetent individuals. However, it can present systemically in the immunocompromised, with a single center retrospective study estimating that of pediatric patients with histoplasmosis in an endemic area, roughly half of immunocompromised patients had disseminated disease affecting the pulmonary, gastrointestinal, integumentary, and nervous systems (1). Organ transplants in particular are a risk for histoplasmosis wherein the first year following transplant is the period of highest risk for histoplasmosis, and given the relative prevalence of kidney transplants, there are multiple case reports of histoplasmosis in renal transplant patients (2, 3).

The differential for histoplasmosis is broad given the large variety of relatively non-specific symptomatology and encompasses a range of infectious, autoimmune, and hematologic/oncologic etiologies as well as system-specific diagnoses (4). As such, a comprehensive evaluation is necessary involving laboratory testing, advanced imaging, and biopsies of affected areas (5). While case reports exist of disseminated histoplasmosis in immunosuppressed patients, to our knowledge none exist in the absence of high-risk exposures (6). We thus present an exceptional case of disseminated histoplasmosis in a renal transplant patient without known exposure to endemic areas highlighting the utility of molecular diagnostics in unclear clinical scenarios.

## Patient information

A 16-year-old male with a history of Bardet-Biedl syndrome and live donor kidney transplant in 2013 for end-stage renal disease due to dysplastic kidneys presented with abdominal pain and diarrhea. For the last one and a half months, he endorsed intermittent generalized abdominal pain often associated with bowel movements, which alternated between constipation and diarrhea. In the days leading to hospitalization, stools became consistently loose and watery. The patient also noted a painless, erythematous papule on his upper abdomen for the past 4 months which had become ulcerated without discharge. His mother reported recent fatigue, anorexia, and approximately ten pounds of weight loss over the past few weeks. He denied fevers, chills, nausea, vomiting, or hematochezia.

Historically, the patient was born in Egypt, lived primarily in Qatar until 2017, then emigrated to California. His renal transplant was performed in Egypt. Travel history included brief travel of up to 2 weeks to New York City, the United Kingdom, and Germany in 2015. Of note, the patient had been receiving treatment for chronic antibody-mediated rejection of his transplant with monthly tocilizumab and IVIG for the previous 6 months. In the week before admission, he had a renal biopsy consistent with worsening rejection. His immunosuppressive regimen was further expanded to include tacrolimus, mycophenolate, and prednisone.

## Clinical findings

**Figure 1:**
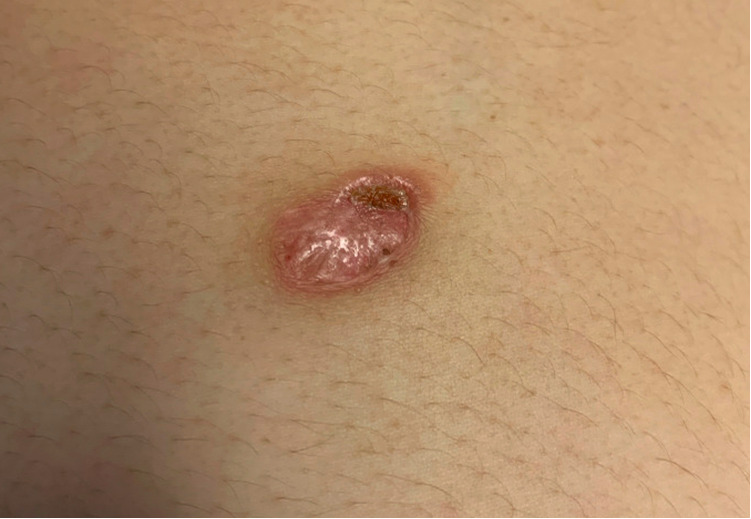
Skin lesion on upper abdomen at the time of presentation.

On presentation, the patient was afebrile with normal vital signs. Physical exam was significant for abdominal distension, and edema noted throughout the right upper extremity and lower extremities bilaterally. Skin lesion was an approximately two- by three-centimeter ovoid plaque with accentuated border and erosion/crusting at the superior aspect (Figure 1A).

## Timeline


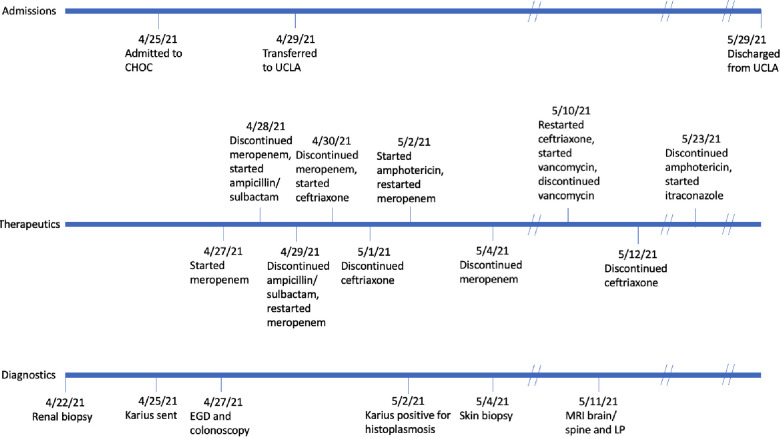


## Diagnostic assessment

**Figure 2:**
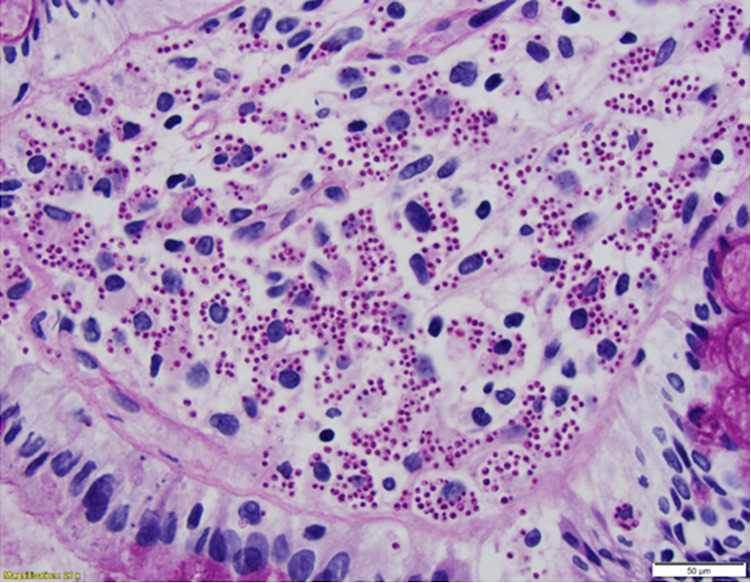
PAS-stained rectal biopsy showing abundant histiocytes within the lamina propria laden with PAS positive organisms.

Labs were significant for WBC 3.26 × 10^3^/μl with 70% neutrophils, 14.4% lymphocytes, and 0.9% eosinophils, platelet count 107 × 10^3^/μl, creatinine 1.31 mg/dl (eGFR 47.7 ml/min/1.73 m^2^), CRP 2.1 mg/dl, CMV DNA PCR was positive but below the 137 copies/ml cutoff, and urinalysis demonstrated 14 WBC/HPF but negative nitrites and leukocyte esterase. Comprehensive stool studies to evaluate for infectious etiologies of diarrhea were unrevealing. Chest x-ray showed patchy retrocardiac opacities, central opacities, opacity along the left lateral thorax, appearance of a widened mediastinum/cardiac silhouette, and soft tissue edema along the right inferior abdominal wall. An echocardiogram demonstrated small posterior and inferior pericardial effusions but no significant regurgitation. An esophagogastroduodenoscopy and colonoscopy demonstrated an erythematous duodenal bulb with villous blunting, erythematous terminal ileum, small ulcerations throughout the colon, and a rectal mass. Biopsies were collected throughout the gastrointestinal (GI) tract and sent for pathology (Figure 2). After the procedure, the patient became febrile to 38.3 °C and developed increased work of breathing concerning for an aspiration pneumonia.

**Figure 3:**
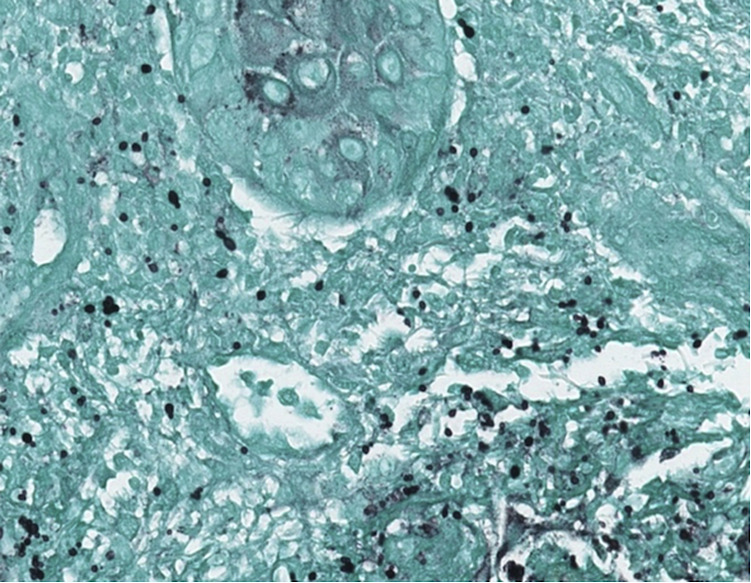
GMS-stained skin biopsy demonstrating numerous budding yeast within the dermis.

The patient subsequently developed abdominal wall swelling affecting his right lower quadrant, and a CT abdomen and pelvis imaging study with contrast revealed an edematous right colon, bibasilar pulmonary infiltrates, mediastinal and mesenteric lymphadenopathy, moderate hydronephrosis of transplanted kidney with perinephric urinoma, hepatosplenomegaly, and extensive edema affecting the adjacent retroperitoneum, mesentery, and abdominal wall. Preliminary GI pathology results were concerning for visceral leishmaniasis but Karius (Karius Inc, Redwood City, CA) molecular testing on blood detected *Histoplasma*. Serum and urine *Histoplasma* antigen testing (MiraVista Diagnostics, Indianapolis, IN) returned positive at 2.34 ng/ml and 5.50 ng/ml, respectively. While fungal tissue culture from his biopsies ultimately returned negative, which could be considered a potential limitation, GI pathology was subsequently reviewed by the Parasitology Branch of the Centers for Disease Control and Prevention who determined the histological appearance was consistent with *Histoplasma* rather than *Leishmania*. Additionally, biopsy of the abdominal skin lesion demonstrated yeast consistent with Histoplasma (Figure 3). Histology revealed epidermal ulceration with underlying granulomatous inflammation with associated necrosis, as well as multiple intra- and extracellular yeast, measuring approximately 2–4 microns with narrow based budding. These findings were confirmed on GMS and PAS special stains. Cerebrospinal fluid (CSF) antigen testing was conducted given headaches and was initially weakly positive. However, repeat testing of CSF IgG and IgM were undetectable twice and CNS histoplasmosis was thought to be unlikely.

Ultimately a diagnosis of disseminated histoplasmosis with gastrointestinal, cutaneous, and probable pulmonary involvement was made. Diagnostic work-up for additional pathogens as part of the differential included tuberculosis, coccidioidomycosis, and *Leishmania* and were all negative. Challenges to the diagnosis included a lack of historical exposure to areas endemic for histoplasmosis and extended duration between symptom onset following transplantation. Additionally, diagnosis was confounded by the need for multiple sampling of tissue specimens to evaluate for pathogenic organisms and disseminated organ loci. Finally, diagnosis was delayed as cultures and specialized antigen/antibody testing required extended turnaround time and advanced processing facilities.

## Therapeutic intervention

Given fever after GI biopsies, the patient was started on meropenem (unknown dosage) empirically on 4/27, which was eventually transitioned to ampicillin/sulbactam (unknown dosage) on 4/28 after serum bacterial cultures did not demonstrate any growth. On transfer to UCLA on 4/29, ampicillin/sulbactam was discontinued and meropenem 1 g every eight hours was initiated and administered from 4/29 to 4/30 and 5/2-5/4 given continued clinical instability. During the course of empiric treatment and prior to diagnosis of histoplasmosis, he also received intermittent ceftriaxone 1 g daily (4/30-5/1, 5/10-5/12) and one dose of vancomycin 750 mg on 5/10. The patient was eventually started on liposomal amphotericin B 4 mg/kg daily, given findings consistent with disseminated histoplasmosis. Twenty-two days of IV liposomal amphotericin B were completed prior to transitioning to oral itraconazole therapy 200 mg twice daily. Itraconazole was increased to 300 mg PO twice daily two months later due to low trough levels; the patient was maintained therapeutic itraconazole levels on this dose thereafter.

## Follow-up and outcomes

Per IDSA guidelines for histoplasmosis treatment monitoring, changes in blood and/or urine antigen levels can be used as indicators for response to therapy. Antigen testing has a sensitivity of 100% in urine and 92.3% in serum with a specificity of 99%. Antigen levels should be measured before treatment is initiated, at two weeks, at one month, every three months during therapy, and for at least six months after treatment is stopped. These markers should also be measured subsequently if treatment failure or relapse is suspected.

Outpatient monitoring by pediatric infectious disease and nephrology teams revealed undetectable serum antigen after four weeks of treatment and detectable urine antigen at lower levels compared to hospitalization. Concurrently, serum itraconazole trough levels and hepatotoxicity were evaluated at regular intervals and adjusted as needed. The regimen was well tolerated with good patient adherence and no adverse effects. He will maintain lifelong treatment with an antifungal agent given his immunosuppressed state and will be periodically monitored for *Histoplasma* disease relapse.

## Discussion

Human histoplasmosis is caused by two varieties of dimorphic fungi: *H capsulatum* var. *capsulatum* and *H capsulatum* var. *duboisii.* In the US, being in an urban metropolitan area like New York City as was the case in our patient is not commonly associated with increased risk of histoplasmosis, which is generally found in the Ohio and Mississippi River Valleys. However, with the use of new immunosuppressive medications and other immunosuppressive conditions, increased spread of histoplasmosis has been reported in states including Texas, New York, Colorado, and California (7). Indeed, serum testing suggests that histoplasmosis is more prevalent worldwide than we would suspect, though its clinical significance is unclear (8). Egypt, where our patient once lived, is not generally recognized as an endemic area for histoplasmosis, with further research needed to address this question. Due to this uncertainty, a potential source of exposure in Egypt cannot be completely ruled out.

Most patients afflicted with histoplasmosis remain asymptomatic throughout the disease course, especially if immunocompetent and otherwise healthy. For immunocompromised patients and pediatric patients, histoplasmosis may present with more acute and severe disease: a study by Garcia-Boyano et al. found that children with HIV diagnosed with disseminated histoplasmosis required prolonged hospitalization (9). If symptomatic, histoplasmosis in pediatric populations predominantly manifests as acute pulmonary histoplasmosis. However, hematogenous spread can result in disseminated disease, and other isolated single-organ infections have been rarely reported including meningeal involvement (4). Signs and symptoms of pulmonary involvement include fever, chest pain, and cough and may include anorexia, weight loss, lymphadenopathy, hepatosplenomegaly, and skin findings. Given these relatively non-specific findings, the differential for histoplasmosis is broad and includes other infectious causes (leishmaniasis, blastomycosis, atypical pneumonias, and tuberculosis), inflammatory (sarcoidosis), and oncologic causes. Indeed, these conditions—especially tuberculosis—can be concurrent in certain endemic areas (10).

Depending on the initial presentation, preliminary laboratory testing may include CBC, CMP, ESR, CRP, procalcitonin, LDH, ferritin, galactomannan, (1-3)-beta-D-glucan, tuberculosis testing, and urine, serum, and CSF testing. Careful radiologic examination should be conducted as appropriate to identify systemic involvement. Of note, common radiologic findings in CNS histoplasmosis include focal mass lesions, diffuse white matter changes, and areas of restricted diffusion (11). Definitive diagnosis includes antigen testing and histologic analysis of tissue cultures, especially blood, liver, skin lesions, CSF, urine, or any other potential sites of involvement (5). In terms of histopathology in histoplasmosis, numerous plasma cells, histiocytes and lymphocytes may be seen with budding yeast forms on H&E staining. Giemsa stain may reveal phagocytic cells containing oval organisms approximately 3–4 microns in diameter with a cap surrounded by a small light halo (12). Periodic Acid Schiff stain demonstrates a similar halo pattern with red-violet coloration of the yeast. In our patient's specific case, leishmaniasis was suspected because of our patient's early travel to Qatar and the similarity in histopathologic features to histoplasmosis, including its small size and intracellular location. However, *Leishmania* can be differentiated from *Histoplasma* by the presence of a dense collection of DNA in the mitochondria known as kinetoplasts (13). More generally, the morphologic and serologic findings seen in this case can potentially be seen in infections by other types of dimorphic fungi, and so definitive diagnosis should be made in conjunction with culture and morphologic, biochemical, mass spectrometry, or nucleic acid studies.

For disseminated histoplasmosis in an immunocompetent host, treatment should include amphotericin B for 2–4 weeks followed by itraconazole for a total of 3 months. In immunosuppressed hosts, long term suppressive therapy may be merited if immunosuppression cannot be discontinued. For CNS involvement, the Infectious Disease Society of America recommends liposomal amphotericin B for 4–6 weeks followed by itraconazole for at least one year or until resolution of CNS symptoms (14). In patients who survive the first month after diagnosis, treatment with an amphotericin formulation followed by an azole for 12 months is usually successful, with only a rare relapse.

We thus present a case of disseminated histoplasmosis in a kidney transplant patient. While there have been previous reports of this in the literature, our case is unique in our patient's underlying genetic condition, travel history, and diagnosis with no known clear exposure to *Histoplasma* endemic areas (2, 15). With the use of novel diagnostic techniques including Karius, serum metagenomics, and next-generation sequencing, we were eventually able to identify histoplasmosis, a feat that would have been difficult with cultures alone (16). A high index of suspicion for fungal infection should thus be maintained in cases of undifferentiated symptoms in immunosuppressed patients, and workup should be comprehensive including laboratory microbiology studies, radiology, and pathology.

## Patient perspective

Patient perspective was unable to be elicited as patient has an intellectual delay.

## Data Availability

The original contributions presented in the study are included in the article/Supplementary Material, further inquiries can be directed to the corresponding author/s.
